# An assessment of an antioxidative status in patients with multiple sclerosis using standard biochemical analyses

**DOI:** 10.5937/jomb0-51901

**Published:** 2025-10-28

**Authors:** Tamara Anđelić, Ivana Stevanović, Mirjana Mijušković, Bratislav Dejanović, Milica Ninković

**Affiliations:** 1 Institute of Medical Biochemistry, Military Medical Academy, Belgrade; 2 Institute of Medical Research, Military Medical Academy, Belgrade, Serbia; 3 Faculty of Medicine, Military Medical Academy, University of Defense, Belgrade; 4 Clinic of Nephrology, Military Medical Academy, Belgrade

**Keywords:** antioxidants, multiple sclerosis, oxidative stress, relapse, remission, antioksidansi, multipla skleroza, oksidativni stres, recidiv, remisija

## Abstract

**Background:**

The study aimed to assess antioxidative status using standard biochemical analyses in multiple sclerosis patients during relapse and remission. Also, to evaluate the effects of gender, drug-modified treatment and clinical characteristics of multiple sclerosis on analyzed antioxidants.

**Methods:**

The study consisted of 178 relapse-remitting multiple sclerosis patients (61 relapse/117 remission), 93 females and 85 males, with a mean age of 40.9±9.8 (average age of 40.9) and 80 matched healthy controls. Ongoing drug-modified treatment received 132 patients. The serum levels of uric acid, total bilirubin, albumin, and transferrin were analyzed in both patients and controls. Extended Disability Status Scale (EDSS), disease duration and annual relapse rate were used as clinical characteristics of multiple sclerosis.

## Abstract (sr)

**Uvod:**

Cilj ove studije bio je određivanje antioksidativnog statusa pacijentima obolelim od multiple skleroze prime nom standardnih biohemijskih testova tokom relapsa i re misije. Istovremeno, cilj studije je bio i da se odredi uticaj pola, lekova koji modifikuju prirodan tok bolesti i kliničkih karakteristika multiple skleroze na analizirane antioksidanse.

**Metode:**

Studija je obuhvatila 178 pacijenata sa relapsno-remitentnom multiplom sklerozom (61 u relapsu/117 u remisiji), 93 žena i 85 muškaraca, 40,9±9,8 godina (prosečna starost 40,9 godina) i 80 zdravih kontrola, uparenih po godinama i polu. 132 pacijenata je primalo terapiju koja modifikuje prirodan tok bolesti. Određene su koncentracije mokraćne kiseline, ukupnog bilirubina, albumina i transferina u serumu pacijenata i kontrolne grupe. Proširena Skala Statusa Onesposobljenosti (EDSS), trajanje bolesti i broj relapsa po godini su predstavljali kliničke karakteristike multiple skleroze.

**Rezultati:**

Antioksidativni status je niži kod obolelih od multiple skleroze u poređenju sa kontrolnom grupom. Nije utvr đena statistički značajna razlika u koncentracijama antioksidanasa tokom relapsa i remisije. Žene u odnosu na muškarce imaju slabiju antioksidativnu zaštitu, koja se ogleda u nižim koncentracijama mokraćne kiseline, ukupnog bili rubina i albumina. Utvrđena je negativna korelacija između trajanja bolesti i vrednosti mokraćne kiseline i između broja relapsa po godini i koncentracije ukupnog bilirubina. Utvrđene su veće vrednosti albumina kod pacijenata koji su na terapiji koja modifikuje prirodan tok bolesti.

**Zaključak:**

U ovoj studiji je dokazana upotreba standardnih biohemijskih analiza za procenu antioksidativne aktivnosti obolelih od multiple skleroze. Pol i kliničke karakteristike bolesti utiču na parametre antioksidativnog statusa i ukazuju na značaj personalizovane, komplementarne, pomoćne antioksidativne terapije u multiploj sklerozi.

## Introduction

Multiple sclerosis (MS) is a heterogeneous, immune-mediated, disabling disease of the central nervous system (CNS) characterized by chronic inflammation, demyelization, and axonal loss [1]. Concerning the course of MS, the disease is divided into three types: relapsing-remitting (RR), primary progressive, and secondary progressive. Relapsingremitting type is the most common MS course in which clearly defined attacks of neurologic symptoms- relapses are followed by periods of partial or complete recovery-remissions [2]. The treatment goal in MS is to avoid relapse occurrence, thus reducing disease activity and preventing disability progression because every relapse attack may leave a permanent neurological deficit [3]. Early treatment initiation with appropriate disease-modifying therapy (DMT) is pivotal for that purpose.

The Expanded Disability Status Scale (EDSS) is a clinical method of quantifying MS disability changes. On the cell level, chronic inflammation, oxidative stress (OS), and blood-brain barrier (BBB) damage happens through time, thus promoting the process of demyelination, disrupting the extent of remyelination, and leading to axonal and neuronal damage [4]. OS is an imbalance between the production of free radicals and the antioxidative capacity to counteract their harmful effects. The cause of OS is either excessive production of free radicals and/or inadequate function of the antioxidant system. Whatever the reason is, the result is extensive oxidative damage to proteins, lipids, and nucleotides, causing their loss of function and degradation [5]. That directly contributes to brain damage. Together with the mitochondrial damage and posterior disturbance of energy metabolism, oxidative stress drives inflammation, neurodegeneration, and axonal degeneration, which give OS a primal role in MS pathogenesis [6] [7]. The CNS is particularly susceptible to OS due to its high energy requirements, limited cell renewal, and increased concentration of OS-prone molecules [8].

Multiple sclerosis is characterized by excessive oxidation, reflected in increased oxidative power [9] and reduced endogenous antioxidant capacity [10]. Enhancing antioxidant potential could be used as a complementary disease-modifying add-on therapy for MS, as suggested by some authors [11] [12] [13]. However, most reported oxidative/antioxidative parameters require sophisticated procedures, robust analytical equipment, and a long delay in reporting the results. On the other hand, routine biochemical analyses are standardized, cost-effective, have a short turnaround time and are widely available in daily clinical practice.

The study aimed to investigate common biochemical analyses, such as uric acid, total bilirubin, albumin, and transferrin, as a screening tool for antioxidative status in MS patients and their levels of relapse and remission. These parameters are involved in various metabolic pathways and, at the same time, have a strong antioxidative capacity [14]. We also intended to evaluate the effect of gender, clinical characteristics and DMT use on the analyzed biochemical parameters.

## Materials and methods

### Patients

One hundred seventy-eight MS patients were recruited from January to December 2017 at the Department of Neurology of the Military Medical Academy in Belgrade, Serbia. The control group consisted of 80 healthy individuals matched in age and gender with the selected patients. The Military Medical Academy Ethics Committee approved the study, which was performed in conformance with the Declaration of Helsinki’s ethical guidelines. Informed consent was obtained from all the participants (patients and controls) before the study.

All patients had a clinically definite diagnosis of MS according to the 2011 McDonald’s criteria [15]. A complete neurological examination with an EDSS assessment was performed [14]. Disease duration was calculated from the onset of the first MS symptoms to entry into the study (expressed in years). The annual relapse rate (ARR) was defined as the total number of relapses divided by the disease duration. Exclusion criteria were primary or secondary progressive MS and other chronic and acute diseases. Based on the clinical presentation, 178 MS patients were divided into the MS relapse (61 subjects) and MS remission groups (117 subjects). The relapse was described as an occurrence of new or worsening of the old symptoms, which lasted more than 24 hours and occurred at least 30 days after a similar event. MS relapse patients were studied at the onset of relapse (within seven days of symptom onset) and before relapse treatment by corticosteroids. Remission patients were relapse-free for at least six months. Based on the current DMT, patients were divided into users and non-users. One hundred ten remission patients (94%) received DMT, as follows: interferons (47 patients), dimethyl-fumarate (28 patients), daclizumab (26 patients), glatiramer acetate (6 patients), hematopoietic stem cell transplantation (2 patient), fingolimod (1 patient). In contrast, only 37% of relapse patients (22) were DMT users who were treated with interferons (8 patients), dimethyl-fumarate (3 patients), fingolimod (3 patients), glatiramer acetate (3 patients), daclizumab (2 patients), hematopoietic stem cell transplantation (2 patients) and mitoxantrone (1 patient). When the study was conducted, daclizumab was still in regular use.

### Biochemical analysis

Venous blood samples were taken from all patients on hospital admission and the control group at regular check-ups. Venipuncture was performed in BD Vacutainer serum tubes (BD Diagnostics, Plymouth, UK). The serum tubes were centrifuged at 3000 rpm for 10 minutes, and a biochemical analysis was performed within 4 hours of venipuncture.

Uric acid is the last compound of purine metabolism. Uric acid concentrations were determinedenzymatically using Siemens reagents on Siemens Advia 1800 Clinical Chemistry System, and results were expressed in μmol/L.

Total bilirubin is a product of hem degradation. The results were analyzed using the diazo method on Siemens Advia 1800 Clinical Chemistry System using Siemens reagents. The results were expressed in μmol/L.

Transferrin binds and transports iron, thus limiting its ability to promote OS. Transferrin forms immune complexes with specific antibodies. The amount of immune complexes was measured immunonephelometrically on a Dade BN-II nephelometer. The results were expressed in g/L.

Albumin is a transport protein. It was determined using the Siemens bromocresol green reagent on the Advia 1800, and the results were expressed in g/L.

### Statistics

The results were presented as means ± standard deviation or median with interquartile range,depending on whether the variables were normally distributed. Categorical data were presented as frequencies. Statistical analysis was performed using GraphPad Prism 5.0 (California, USA). Statistical significance between two measured continuous variables was determined using the Mann–Whitney or the unpaired Student t-test. The relationship between the variables was tested using the non-parametric Spearman correlation. The Chi-Square test analyzed categorical data. Values of p less than 0.05 were considered significant.

## Results

### Clinical characteristics of MS patients

The basic demographic and clinical characteristics of the MS patients are shown in Table 1. No statistically significant difference in age and gender was found between MS patients and controls (p=0.601; p=1.0). Patients with relapse had a significantly higher EDSS (p<0.001), a higher disease duration and annual relapse rate (p<0.001; p<0.01), and a lower number of DMT users (p<0.001) compared to patients in clinical remission.

**Table 1:** Demographic and clinical characteristics of MS patients and healthy controls. Data are expressed as mean ± standard deviations (x±SD) or median (25th–75th percentile). Categorical variables are presented as relative frequencies.

Characteristic	MS Patients	MS Patients-Relapse	MS Patients-Remission	Healthy Controls
Number of subjects	178	61	117	80
Sex (female/male)	93/85	37/24	56/61	44/36
Age (years)	40.9±9.8	39.2±10.9	41.9±9.1	39.3±9.0
EDSS	2.5 (1.5–4.1)	3.5 (2.5–5.0)	2.0 (1.0–3.0)	NA
Disease duration (years)	8.0 (5.0–13.0)	9.0 (5.0–14.0)	6.0 (3.0–10.5)	NA
Annual relapse rate	0.3 (0.2–0.6)	0.5 (0.3–1.3)	0.3 (0.2–0.4)	NA
Patients on DMT	132	22	110	NA

### Antioxidant status in MS patients

Serum antioxidant levels were analyzed between MS patients and healthy controls and between MS patients in relapse and remission. Uric acid, total bilirubin, transferrin, and albumin were significantly decreased in MS patients compared to healthy controls (Table 2). There was no statistically significant difference in antioxidant parameters between MS patients in relapse and remission (Table 2).

**Table 2:** Antioxidant status in the whole MS patients, healthy controls, MS patients in relapse and MS patients in remission. Data are presented as median (25th–75th percentile). P-values; Mann-Whitney test

Parameter	MS Patients	Healthy Controls	p	MS Patients–Relapse	MS Patients–Remission	p
Uric acid (mmol/L)	261 (215–307)	295 (229–349)	<0.01	257 (241–274)	270 (257–283)	0.575
Total Bilirubin<br>(mmol/L)	10 (7–12)	12 (8–16)	<0.001	9 (7–12)	10 (7–12)	0.291
Transferrin (g/L)	2.6 (2.2–2.9)	2.7 (2.4–3.1)	<0.05	2.5 (2.2–2.9)	2.6 (2.3–2.9)	0.149
Albumin (g/L)	45 (43–47)	48 (44–49)	<0.001	44 (41–46)	45 (43–47)	0.061

### Gender effect on antioxidant status in MS

We also investigated the possible effects of gender on antioxidant status in the MS, relapse and remission group. After the Chi-Square test (data not shown), no differences were found in female and male subgroups regarding age, EDSS, disease duration, ARR or DMT use. Female subpopulations had statistically significantly lower uric acid, albumin and total bilirubin levels in all analyzed groups (total MS, remission, relapse, and controls). Transferrin levels were significantly higher in women than in men (total MS, remission and relapse groups), while the transferrin comparison in controls did not reach statistical significance (Table 3).

**Table 3:** Gender-specific differences in antioxidant status in the whole MS patients, MS patients in relapse, MS patients in remission and healthy controls. Data are presented as mean ± standard deviations (x̄±SD) or median (25th–75th percentile). P-values: Mann-Whitney test was used for all analyses except for transferrin (relapse, remission and controls) and uric acid (relapse), where an unpaired Student’s t-test was used.

	MS Patients			MS Patients–Relapse			MS Patients–Remission			Healthy Controls		
	Females	Males	p	Females	Males	p	Females	Males	p	Females	Males	p
Uric acid<br>(mmol/L)	232<br>(196–270)	297<br>(265–370)	<0.001	240±55	291±80	<0.001	228<br>(196–268)	304<br>(266–380)	<0.001	235<br>(211–263)	340<br>(316–370)	<0.001
Total<br>Bilirubin	8<br>(7–10)	11<br>(8–15)	<0.001	8<br>(7–10)	10<br>(7–16)	<0.05	8<br>(7–10)	11<br>(10–15)	<0.001	9<br>(7–12)	14<br>(11–19)	<0.001
Transferrine<br>(g/L)	2.7<br>(2.3–3.0)	2.4<br>(2.2–2.8)	< 0.01	2.6±0.4	2.4±0.4	<0.01	2.7±0.5	2.5±0.4	<0.05	2.8 ±0.5	2.6±0.5	<0.05
Albumin<br>(g/L)	44<br>(41–46)	46<br>(44–47)	<0.001	44<br>(40–46)	46<br>(44–47)	<0.05	44<br>(41–46)	47<br>(45–49)	<0.001	45<br>(43–49)	48<br>(46–50)	

### Effect of DMT on antioxidant status

The analysis of MS patients based on their DMT use showed that 132 patients were treated with DMT, while 46 of them did not receive MS therapy. There was no statistically significant difference in terms of age (p=0.550), gender (p=0.327), EDSS (p=0.135), or disease duration (p= 0.174) between the two analyzed groups. After performing statistical analysis, we found a significant difference in albumin concentration, with higher albumin levels in the patients treated with DMT compared to the patients without DMT (45 g/L vs. 43 g/L, p<0.05) (Table 4).

**Table 4:** Comparisons of MS patients with and without DMT use. Data are presented as median (25th–75th percentile). p values; Mann-Whitney test

Parameter	MS patients on DMT	MS patients without DMT	p-value
Uric acid (mmol/L)	264 (216–305)	253 (210–309)	0.426
Total biirubin (mmol/L)	10 (7–13)	9 (7–11)	0.319
Transferrin (g/L)	2.6 (2.2–2.9)	2.5 (2.3–2.9)	0.516
Albumin (g/L)	45 (43–47)	43 (41–46)	<0.05

### Relations between antioxidant status and clinical characteristics of MS

In the whole MS group, disease duration negatively correlated with uric acid (r=-0.220, p<0.05<), while the annual relapse rate negatively correlated with total bilirubin (r=-0.188, p<0.05). We found no correlation between EDSS and antioxidants.

In relapsing MS patients, annual relapse rate and total bilirubin were negatively correlated (r=- 0.274, p<0.05), while no correlations were found between antioxidants and disease duration or EDSS.

In patients with MS remission, we only found a negative correlation between disease duration and uric acid (r=-0.187, p<0.05), while correlations between antioxidants and ARR, or EDSS, did not reach statistical significance (data not shown).

## Discussion

The most important result of the study is that the antioxidants, uric acid, total bilirubin, transferrin, and albumin are significantly lower in MS patients than in control subjects. Both in remission and relapse, an antioxidant status was reduced in MS patients compared to the healthy group. This reflects a reduced antioxidant capacity in MS due to excessive and long-term oxidative damage.

Uric acid is a strong natural scavenger of reactive oxygen and nitrogen species, especially in the CNS [15] [16] [17]. It is still not elucidated whether decreased serum uric acid levels in MS, reported in this study, as in the studies by other authors [18] [19], are the cause or the consequence of the disease. However, its potential use as a biomarker of MS activity and progression is well known. The negative correlation of uric acid and disease duration showed in this study, as in the study by Moccia et al. [20], indicates the progressive loss of antioxidant residue power during the MS course. This progressive antioxidant deterioration is also reflected in a negative correlation between total bilirubin and ARR, demonstrated in this study, with lower bilirubin values in patients with more active MS. Total bilirubin inhibits superoxide anion production, suppresses phospholipids and proteins peroxidation, and protects immune and nerve system, both compromised in the MS [21]. Today, bilirubin is well known for its strong antiinflammatory and immune suppressive activities [22].

Albumin and transferrin are negative molecules of the acute phase response. Inflammation is a major feature of MS; therefore, lower albumin and transferrin levels are expected as inflammation leads to OS and vice-versa. Both molecules are transport proteins. While albumin binds and delivers various ligands (drugs, fatty acids, bilirubin), transferrin is only responsible for iron molecules [23]. Its antioxidant effect prevents the Fenton and Heber-Weiss reaction (by binding iron and/or copper), preventing reactive oxygen species formation [24]. Transferrin also accelerates myelin biosynthesis in oligodendrocytes, thus inducing the process of remyelination [25]. This also contributes to the reduced transferrin in MS as an attempt to compensate for myelin loss and delay the disease progression. While lower serum albumin is well documented in MS [26] [27], only a few studies have analyzed transferrin [10] [28]. Transferrin is, despite reduced antioxidant defence, reduced due to a higher proportion of women in the MS [10], as higher transferrin characterized to females means lower iron. An almost equal number of female and male participants in this study resulted from dominantly military-insured males in Serbia, whose host hospital is the Military Medical Academy.

In our previous study, OS in MS represented increased malondialdehid, nitric oxide, and prooxidant-antioxidant balance. In these circumstances of high OS, antioxidant potential was documented by uric acid, and total bilirubin, transferrine, and albumin were decreased [2]. For example, most substances, like uric acid, can be prooxidants and antioxidants, depending on the location or dose [17].

The second important finding in this study is the lack of difference in antioxidant status between remission and relapse patients. This indicates persistent oxidative stress and permanent CNS damage during MS, regardless of the clinical presentation. Even if the disease remains in clinical remission, the body’s antioxidants have to cope with many reactive species, meaning clinical remission is only the top of the iceberg that does not reflect the ongoing inflammation. While inflammation is usually connected with relapses and neurodegeneration with progression, both pathologies are present during the whole MS phase. To prevent further oxidative damage and preserve natural antioxidant capacity, early and effective antioxidant treatment in MS is beneficial, with particular attention to gender differences.

Regarding gender differences, MS is a female predominant disease, with a more severe course in males due to immune and nervous system differences, hormones, and genetic changes. The only antioxidant in this study that was lower in male participants than in female participants was transferrin. Burger et al. also reported a lower transferrin concentration in males [29]. The results of this study demonstrated significantly higher levels of uric acid, total bilirubin, and albumin in males vs females. Estrogen in females contributes to higher renal uric acid clearance [30], while lower haemoglobin results in less bilirubin degradation. The results on sex-specific differences in serum albumin concentrations are vague. Lower albumin in females is due to differences in hormone status, as oral contraceptive use leads to lower albumin levels [31]. Albumin also reflects nutritional status, so differences in dietary habits also contribute to higher albumin levels in men [32]. Albumin is also a transport molecule responsible for the transfer of bilirubin. Many authors have reported that lower bilirubin concentrations in MS [33] [34] [35] reflect the constant battle against OS attacks. This fight also resulted in lower uric acid levels in MS patients [20]. Uric acid is a natural peroxynitrite scavenger that neutralizes the overproduction of peroxynitrite. Unlike the results of this study, some researchers reported a link between uric acid and MS clinical activity [36] [37].

Whether the reduced antioxidant status in MS is a primary or secondary deficit related to the fight against OS is not yet clear. We believe that reduced antioxidant status is more likely a secondary epiphenomenon, which is consistent with other researchers [38] [39]. This concept is important for the development of therapeutic strategies. Improving antioxidant defences in MS and effective additive antioxidant treatment could reduce the severity of the disease or improve its marked progression. The introduction of dietary prophylaxis could also be a strategy to reduce OS.

After dividing MS patients by DMT use, we found increased levels of all analyzed antioxidants, with albumin reaching a statistical significance in the DMT users’ group. DMT modify the course of MS through suppression or modulation of immune function. These drugs are designed to slow the MS progression and reduce the frequency and severity of relapses. Through inflammation and immune suppression, DMT also promotes antioxidative effects. Dimethyl and mono methyl fumarate are the only MS-approved drugs with directly redox-modulating properties. The beneficial DMT effects on OS in MS are well known [40], as well as improved recovery from relapse-induced disability with ongoing DMT [2]. Theoretically, combining DMT and antioxidant supplementation may act synergistically, resulting in a more powerful treatment. Importantly, applying antioxidants requires both great caution and excellent knowledge. Caution due to interactions between antioxidants and DMT, which reduce DMT levels and thus aggravate MS symptoms [41] and knowledge due to tissue damage in MS is restricted to the CNS, meaning that selected antioxidants should pass through the BBB.

## Conclusion

In conclusion, standard biochemical analyses could be used to assess antioxidative status in patients with MS, both in relapse and remission. This could be both an accurate and cheap screening tool for antioxidant supplementation- a promising therapeutic goal and add-on MS therapy.

## Dodatak

### Acknowledgements

I appreciate the late Prof. Dragana Obradovic, who provided guidance, support, and true inspiration. This work was supported by the project MFVMA 02/24-26 of the University of Defense of the Republic of Serbia.

### Conflict of interest statement

All the authors declare that they have no conflict of interest in this work.

## References

[1] Oh J, Vidal-Jordana A, Montalban X (2018). Multiple sclerosis: Clinical aspects. Curr Opin Neurol.

[2] Obradović D, Anđelić T, Ninković M, Dejanović B, Kotur-Stevuljević J (2021). Superoxide dismutase (SOD), advanced oxidation protein products (AOPP), and disease-modifying treatment are related to better relapse recovery after corticosteroid treatment in multiple sclerosis. Neurol Sci.

[3] Goodin D S, Reder A T, Bermel R A, Cutter G R, Fox R J, John G R, Lublin F D, Lucchinetti C F, Miller A E, Pelletier D, Racke M K, Trapp B D, Vartanian T, Waubant E (2016). Relapses in multiple sclerosis: Relationship to disability. Mult Scler Relat Disord.

[4] Sladojević M, Nikolić S, Živanović Ž, Simić S, Sakalaš L, Spasić I, Ilinčić B, Čabarkapa V (2024). Determination of systemic inflammatory biomarkers in multiple sclerosis. J Med Biochem.

[5] Singh A, Kukreti R, Saso L, Kukreti S (2019). Oxidative Stress: A Key Modulator in Neurodegenerative Diseases. Molecules.

[6] Lassmann H, van Horssen J (2016). Oxidative stress and its impact on neurons and glia in multiple sclerosis lesions. Biochim Biophys Acta Mol Basis Dis.

[7] Pegoretti V, Swanson K A, Bethea J R, Probert L, Eisel U L M, Fischer R (2020). Inflammation and Oxidative Stress in Multiple Sclerosis: Consequences for Therapy Development. Oxid Med Cell Longev.

[8] Cobley J N, Fiorello M L, Bailey D M (2018). 13 reasons why the brain is susceptible to oxidative stress. Redox Biol.

[9] Ayala A, Muñoz M F, Argüelles S (2014). Lipid Peroxidation: Production, Metabolism, and Signaling Mechanisms of Malondialdehyde and 4-Hydroxy-2-Nonenal. Oxid Med Cell Longev.

[10] Siotto M, Filippi M M, Simonelli I, Landi D, Ghazaryan A, Vollaro S, Ventriglia M, Pasqualetti P, Rongioletti M C A, Squitti R, Vernieri F (2019). Oxidative Stress Related to Iron Metabolism in Relapsing Remitting Multiple Sclerosis Patients With Low Disability. Front Neurosci.

[11] Miller E D, Dziedzic A, Saluk-Bijak J, Bijak M (2019). A Review of Various Antioxidant Compounds and their Potential Utility as Complementary Therapy in Multiple Sclerosis. Nutrients.

[12] Emamgholipour S, Hossein-Nezhad A, Sahraian M A, Askarisadr F, Ansari M (2016). Evidence for possible role of melatonin in reducing oxidative stress in multiple sclerosis through its effect on SIRT1 and antioxidant enzymes. Life Sci.

[13] Miller E, Wachowicz B (2013). Advances in Antioxidative Therapy of Multiple Sclerosis. Curr Med Chem.

[14] Yang D, Su Z, Wu S, Bi Y, Li X, Li J, Lou K, Zhang H, Zhang X (2016). Low antioxidant status of serum bilirubin, uric acid, albumin and creatinine in patients with myasthenia gravis. Int J Neurosci.

[15] Polman C H, Reingold S C, Banwell B, Clanet M, Cohen J A, Filippi M, Fujihara K, Havrdova E, Hutchinson M, Kappos L, Lublin F D, Montalban X, O'Connor P, Sandberg W M (2011). Diagnostic criteria for multiple sclerosis: 2010 Revisions to the McDonald criteria. Ann Neurol.

[16] Kurtzke J F (1983). Rating neurologic impairment in multiple sclerosis: An expanded disability status scale (EDSS). Neurology.

[17] Sautin Y Y, Johnson R J (2008). Uric Acid: The Oxidant-Antioxidant Paradox. Nucleosides Nucleotides Nucleic Acids.

[18] Wang L, Hu W, Wang J, Qian W, Xiao H (2016). Low serum uric acid levels in patients with multiple sclerosis and neuromyelitis optica: An updated meta-analysis. Mult Scler Relat Disord.

[19] Niu P, Song B, Wang X, Xu Y (2020). Serum Uric Acid Level and Multiple Sclerosis: A Mendelian Randomization Study. Front Genet.

[20] Moccia M, Lanzillo R, Costabile T, Russo C, Carotenuto A, Sasso G, Postiglione E, de Luca P C, Vastola M, Maniscalco G T, Palladino R, Brescia M V (2015). Uric acid in relapsing-remitting multiple sclerosis: A 2-year longitudinal study. J Neurol.

[21] Stocker R (2004). Antioxidant Activities of Bile Pigments. Antioxid Redox Signal.

[22] Juping D, Yuan Y, Shiyong C, Jun L, Xiuxiu Z, Haijian Y, Jianfeng S, Bo S (2017). Serum bilirubin and the risk of rheumatoid arthritis. J Clin Lab Anal.

[23] Moos T, Nielsen T R, Skjorringe T, Morgan E H (2007). Iron Trafficking Inside the Brain. J Neurochem.

[24] Dopsaj V, Martinović J, Dopsaj M, Stevuljević J K, Bogavac-Stanojević N (2011). Gender-Specific Oxidative Stress Parameters. Int J Sports Med.

[25] Franco P G, Pasquini L A, Pérez M J, Rosato-Siri M V, Silvestroff L, Pasquini J M (2015). Paving the way for adequate myelination: The contribution of galectin-3, transferrin and iron. FEBS Lett.

[26] Aydin O, Ellidag H Y, Eren E, Kurtulus F, Yaman A, Yilmaz N (2014). Ischemia modified albumin is an indicator of oxidative stress in multiple sclerosis. Biochem Med (Zagreb).

[27] Oliveira S R, Kallaur A P, Reiche E M V, Kaimen-Maciel D R, Panis C, Lozovoy M A B, Morimoto H K, Maes M, Dichi I, Simão A N C (2017). Albumin and Protein Oxidation are Predictors that Differentiate Relapsing-Remitting from Progressive Clinical Forms of Multiple Sclerosis. Mol Neurobiol.

[28] Khalil M, Riedlbauer B, Langkammer C, Enzinger C, Ropele S, Stojakovic T, Scharnagl H, Culea V, Petzold A, Teunissen C E, Archelos J, Fuchs S, Fazekas F (2014). Cerebrospinal fluid transferrin levels are reduced in patients with early multiple sclerosis. Mult Scler.

[29] Burger A, Kotze M J, Stein D J, van Rensburg S J, Howells F M (2020). The relationship between measurement of in vivo brain glutamate and markers of iron metabolism: A proton magnetic resonance spectroscopy study in healthy adults. Eur J Neurosci.

[30] Mateos A F, García P J, Ramos T, González P, Ordás J (1986). Sex differences in uric acid metabolism in adults: Evidence for a lack of influence of estradiol-17b (E2) on the renal handling of urate. Metabolism.

[31] Naz F, Jyoti S, Akhtar N, Siddique Y H, Rahul (2016). Effect of Oral Contraceptive Pills on the Blood Serum Enzymes and DNA Damage in Lymphocytes Among Users. Indian J Clin Biochem.

[32] Weaving G, Batstone G F, Jones R G (2016). Age and sex variation in serum albumin concentration: An observational study. Ann Clin Biochem.

[33] Peng F, Deng X, Yu Y, Chen X, Shen L, Zhong X, et al (2011). Serum Bilirubin Concentrations and Multiple Sclerosis. J Clin Neurosci.

[34] Kapitulnik J (2004). Bilirubin: An Endogenous Product of Heme Degradation with Both Cytotoxic and Cytoprotective Properties. Mol Pharmacol.

[35] Chen J, Tu Y, Connolly E, Ronnett G (2005). Heme Oxygenase-2 Protects Against Glutathione Depletion-induced Neuronal Apoptosis Mediated by Bilirubin and Cyclic GMP. Curr Neurovasc Res.

[36] Sotgiu S, Pugliatti M, Sanna A, Sotgiu A, Fois M L, Arru G, Rosati G (2002). Serum uric acid and multiple sclerosis. Neurol Sci.

[37] Guerrero A L, Gutiérrez F, Iglesias F, Martín-Polo J, Merino S, Martín-Serradilla J I, Laherrán E, Tejero M A (2011). Serum uric acid levels in multiple sclerosis patients inversely correlate with disability. Neurol Sci.

[38] Pakpoor J, Seminog O O, Ramagopalan S V, Goldacre M J (2015). Clinical associations between gout and multiple sclerosis, Parkinson's disease and motor neuron disease: Record-linkage studies. BMC Neurol.

[39] Chittoor G, Kent J W, Almeida M, Puppala S, Farook V S, Cole S A, Haack K, Göring H H H, Maccluer J W, Curran J E, Carless M A, Johnson M P, Moses E K, Almasy L, Mahaney M C (2016). GWAS and transcriptional analysis prioritize ITPR1 and CNTN4 for a serum uric acid 3p26 QTL in Mexican Americans. BMC Genomics.

[40] Topić A, Vasić M, Marković B, Milinković N, Dinčić E (2022). The Effects of Disease-Modifying Therapies on Oxidative Stress in Patients With Relapsing-Remitting Multiple Sclerosis. Clin Neuropharmacol.

[41] Tobore T O (2021). Oxidative/Nitroxidative Stress and Multiple Sclerosis. J Mol Neurosci.

